# Notes and News

**Published:** 1904-12-03

**Authors:** 


### NOTES AND NEWS.

DURING his recent visit to Chichester the King paid a visit to King Edward VII.'s Sanatorium in course of erection at Midhurst.

AN outbreak of typhoid fever has occurred in the Cork District Lunatic Asylum.

A NEW nurses' home has been opened at Rochdale as a memorial to Queen Victoria.

BARON NATHANIEL DE ROTHSCHILD is stated to have given £80,000 for the extension of the Rothschild Hospital in Vienna.

SIR HENRY BURDETT will deliver an address on behalf of the League of Mercy at the Grand Pavilion, Broadstairs, on Thursday, December 8th.

THE late Mr. A. N. Dodds, of Tynemouth, has bequeathed £500 to the Grange-over-Sands Convalescent Home, of which he was one of the founders.

THE North Charitable Infirmary, Cork, has received a gift of £1,000 from Lieut.-Colonel O'Mahony McDonnell, M.D., for the endowment of a bed in the institution.

THE London District Committee of the National Amalgamated Society of Operative House and Ship Decorators appealed to the Metropolitan Asylums Board to rescind the regulation requiring painters employed by them to be vaccinated. The appeal, which was coupled with another request, has not been complied with.

IN the death of Dr. George Vivian Poore sanitary science has lost one of its most eloquent exponents. He was an able physician and interesting personality. He was consulting physician to University College Hospital, where he also received his medical training. He was at one time domestic physician to the late Prince Leopold. In sanitary matters he held very definite views, especially on sewage disposal, and he wrote some useful works of a popular and practical character on the subject.

A NEW infectious diseases hospital has been opened at Lincoln. Each of the wards is 36 feet long by 26 feet wide, and will contain six beds.

THE Nottingham and Notts Sanatorium, at which Lady Victoria Manners recently opened a ward to the memory of Lord Edward Manners, now contains 30 beds. The Duke of Portland presented the site.

A SESSIONAL meeting of the Royal Sanitary Institute was held on November 26th at Nottingham, when members and visitors inspected the Bacteriological and Chemical Laboratory, destructor works, and sanitary wharves.

THE Hamburg-American Steamship Company are fitting up one of their ships for a cruise in the Mediterranean next May as a floating sanatorium. This will only be available for those who can pay a good price for the accommodation.

AN appeal for funds is being made by the National Society for the Prevention of Tuberculosis. We hope the appeal will be successful. The public possibly do not realise how much power for good such an association can yield. By keeping the records of the various movements towards the prevention and cure of consumption in one centre; by providing useful information when any new institution is proposed, and by encouraging uniformity in the methods of recording the experience gained in sanatoria all over the country, the Society can render immense service to the community and to medical science all over the world.

AT the Exhibition of Indian and Colonial products to be held next year at the Crystal Palace, an endeavour will be made to bring together, as far as possible, a complete collection of the Indian and Colonial drugs and pharmaceutical preparations which are contained in the Addendum to the British Pharmacopœia, or which are likely to be included in the "Imperial" Pharmacopœia which the General Medical Council is believed to have in contemplation. Many of these drugs are scarcely at all known to British pharmacists, and it is at least possible that in certain cases some of them may offer advantages worthy of being secured, either for direct administration or as sources of alkaloids. The original intention of the Addendum was merely to authorise the substitution, in certain localities, of indigenous substitutes for some of the ordinary officinal ingredients of certain preparations, *e.g.*, of some of the native fixed vegetable oils for olive oil in ointments, and so forth; or to modify the composition of preparations with reference to the temperature of the locality in which they were to be kept and employed; but it is manifest that a real utilisation of the pharmaceutical products of the empire might be carried much further than this, and might tend to replace "foreign" by "native" products in many instances. The controllers of the exhibition will be well advised in seeking to render this branch of it as complete as possible, and it is to be hoped that their labours in this direction will be appreciated by pharmaceutical authorities at home.

